# ESTRO consensus recommendations: target volume and organ at risk delineation in rectal endoluminal high dose rate brachytherapy

**DOI:** 10.1016/j.ctro.2026.101341

**Published:** 2026-10-31

**Authors:** A.C. Valkenburg, E.J. Van Limbergen, J. Knoth, B. Fionda, K. Neelis, C.S.E.W. Schuurhuizen, M.A. Gambacorta, R. Krishnatry, B. van Triest, A. Appelt, R. Sripadam, A.S. Myint, T. Vuong, M. Bilski, N. Tselis, L. Varela Cagetti, M.P. Schmid, R. Engineer, V. Vendrely, R. Nout, A. Żółciak-Siwińska, P. Hass, F. Bakers, C. Kirisits, P. Cisek, O. Coen, A. Gilbert, C. Brooker, H. Dapper, R. Schmid, B.J.P. Hupkens, A.J. Stewart, M. Berbée, On behalf of GEC-ESTRO

**Affiliations:** a Department of Radiation Oncology (Maastro), GROW Research Institute for Oncology and Reproduction, Maastricht University Medical Centre+, Maastricht, the Netherlands; b Medical University/General Hospital of Vienna, Department of Radiation Oncology, Comprehensive Cancer Center, Vienna, Austria; c Fondazione Policlinico Universitario Agostino Gemelli IRCCS, Rome, Italy; d Department of Radiotherapy, Leiden University Medical Center, Leiden, the Netherlands; e Department of Radiotherapy, Erasmus MC Cancer Institute, University Medical Center Rotterdam, the Netherlands; f Università Cattolica Del Sacro Cuore, Rome, Italy; g Department of Radiation Oncology, Tata Memorial Centre, Mumbai, India; h HBNI University, Mumbai, India; i Department of Radiation Oncology, NKI-AVL, Amsterdam, the Netherlands; j Department of Oncology, Section of Radiotherapy, Rigshospitalet - Technical University Hospital of Greater Copenhagen, Copenhagen, Denmark.; k Department of Health Technology, Technical University of Denmark, Copenhagen, Denmark.; l The Clatterbridge Cancer Centre NHS Foundation Trust, Liverpool, UK; m Clatterbridge Centre for Oncology, Dept of Molecular & Cancer Medicine, Liverpool, UK; n University of Liverpool, UK; o Jewish General Hospital, Montreal, Canada; p McGill University, Montreal, Canada; q Radiotherapy Department, Affidea Nu-med Center of Oncological Diagnostics and Therapy, Zamość, Poland; r Department of Radiotherapy of Oncology, University of Frankfurt, Germany; s Institut Paoli-Calmettes, France; t Department of radiation oncology, CHU de Bordeaux, Bordeaux, France; u Brachytherapy Department, Maria Sklodowska-Curie National Research Institute of Oncology, Roentgena 5, 02-781 Warszaw, Poland; v Department of Radiooncology, Helios Klinikum Erfurt, University Campus of Health and Medical University, Erfurt, Germany; w Department of radiology and nuclear medicine, Maastricht University Medical Centre+, Maastricht, the Netherlands; x Brachytherapy Department, Saint John's Cancer Center, Lublin, Poland.; y Clinics of Radiotherapy, Medical University of Lublin, Poland; z Leeds Cancer Centre, Leeds Teaching Hospitals NHS Trust, Leeds, UK; aa Leeds Institute of Medical Research, University of Leeds, Leeds, UK; ab Royal Surrey Hospital, Guildford, UK; ac Department of Radiation Oncology, Cyberknife and Radiation Therapy, Center for Integrated Oncology Aachen Bonn Cologne Düsseldorf (CIO ABCD), Faculty of Medicine and University Hospital of Cologne, University of Cologne, 50937 Cologne, Germany.; ad University of Surrey, Guildford, UK

**Keywords:** Consensus, Brachytherapy, Radiotherapy, Rectal Neoplasms, Organ preservation

## Abstract

**Background and purpose:**

Lack of international guidelines is hampering treatment quality and progress in the field of rectal endoluminal high dose rate brachytherapy (EHB). Standardisation of dose reporting would allow for better comparison of clinical results between patient cohorts and allow tumour control and normal tissue complication probability modelling. This work presents consensus recommendations for target volume and organs at risk (OAR) delineation in rectal EHB to support standardised dose reporting, for which consistent delineation is a prerequisite.

**Materials and methods:**

Recommendations were developed according to the AGREE II instrument, using a modified Delphi approach. The process included selection of international experts, a survey on current practices, delineation of three patient cases, and three consensus meetings to discuss the findings and formulate recommendations.

**Results:**

The expert group included 30 members from nine countries. The survey and first delineation round showed marked variation in treatment preparation and in the definition and delineation of target volumes and OARs. Consensus was reached on prerequisites for adequate delineation. Target volumes for dose reporting were defined: GTV primary at the time of EHB (GTVp_BT), an evaluation structure (GTVp_BT_ev05), and any macroscopic lymph nodes at the time of EHB (GTVn_BT). Recommendations for OAR selection and delineation were also established.

**Conclusion:**

The GI GEC working group of GEC-ESTRO has developed consensus recommendations for target volume definition and OAR delineation in rectal EHB. These recommendations will be implemented in clinical practice. The proposed structures provide a foundation for standardised dose reporting, independent of local prescription practices.

## Highlights

• Comparison of clinical results is essential for progress in rectal brachytherapy.
• Non-uniform dose reporting in rectal brachytherapy hampers comparison of outcomes.
• International consensus on target volume and OAR contouring in rectal brachytherapy.
• Uniform delineation will enable standardisation of dose reporting.

## 1 Introduction

Colorectal cancer ranks as the 3rd most prevalent cancer worldwide [1]. The therapeutic paradigm for rectal cancer has progressively evolved towards organ preserving approaches [2], [3]. These offer selected patients the opportunity to avoid the substantial morbidity associated with radical resection, thereby preserving anorectal function and enhancing quality of life.

Complete response after radiotherapy enables organ preservation. Various strategies have been explored to improve the chance of a complete response, including dose escalation [4]. Dose escalation through external beam radiotherapy (EBRT) irradiates a larger volume of surrounding normal tissue, increasing the risk of toxicity as doses increase. Endorectal brachytherapy (also known as interventional radiotherapy) has emerged as a promising modality to deliver higher doses to the residual tumour, as the steep dose fall-off enables more selective dose delivery. This allows for dose escalation to the tumour and better sparing of the surrounding organs at risk (OAR). Randomised trials and prospective series have reported favourable oncological outcomes, including excellent local tumour control, through radiotherapy dose escalation using endorectal brachytherapy as part of organ-preserving treatment protocols [5], [6], [7], [8], [9], [10]. Consequently, organ preservation is increasingly recognised and endorsed in both national and international clinical guidelines as a viable, oncologically sound alternative to conventional surgical approaches and prior standard-of-care therapies [11], [12], [13], [14].

Although endorectal brachytherapy can be a valuable treatment option, it is not commonly used in clinical practice due to a lack of treatment machines, the complexity of the procedure, the lack of treatment guidelines and limited high-quality data on local control and toxicity. Verrijssen et al. systematically reviewed radiation toxicity after various endorectal brachytherapy techniques, evaluating 38 studies with 3682 patients [15]. They noted that few specific conclusions could be drawn regarding dose-related toxicity risk for the various techniques due to non-uniform reporting strategies.

Standardisation of dose reporting and treatment data would allow for better comparison of clinical results between patient cohorts, facilitate pooled analyses and future multicentre trials, and enable normal tissue complication probability (NTCP) and tumour control probability (TCP) modelling. Adequate NTCP and TCP models are essential to fully understand the relationship between delivered dose, local control and toxicity, and to further optimise treatment guidelines, thereby improving treatment quality and progress in the field of endorectal brachytherapy.

The Groupe Européen de Curiethérapie-European Society for Radiotherapy and Oncology (GEC-ESTRO) published consensus recommendations for endorectal radiation using contact X-ray brachytherapy (CXB) in 2022. Moreover, *ESTRO consensus guidelines on GTV delineation and dose-escalation in rectal cancer* were published in May 2026 [16]. However, consensus recommendations specifically for image-based rectal endoluminal high dose rate brachytherapy (EHB) do not yet exist. A prerequisite for standardised dose reporting in this setting is standardisation of both target volume and OAR delineation.

The current project aims to reach consensus on target volume definition and OAR delineation in rectal EHB. This will enable standardised reporting and form the foundation for a second set of consensus recommendations addressing patient selection, treatment preparation, dose prescription, treatment planning, dose reporting, and follow-up in rectal EHB.

## 2 Methods and materials

The recommendations were developed according to the AGREE II instrument [17] using a modified Delphi approach. No formal quantitative consensus threshold was prospectively defined; instead, consensus was achieved through iterative discussion and refinement of the recommendations during expert group meetings and was considered reached when no objections remained among the participating experts. The development consisted of eight steps (Fig. 1):

**Fig. 1:**
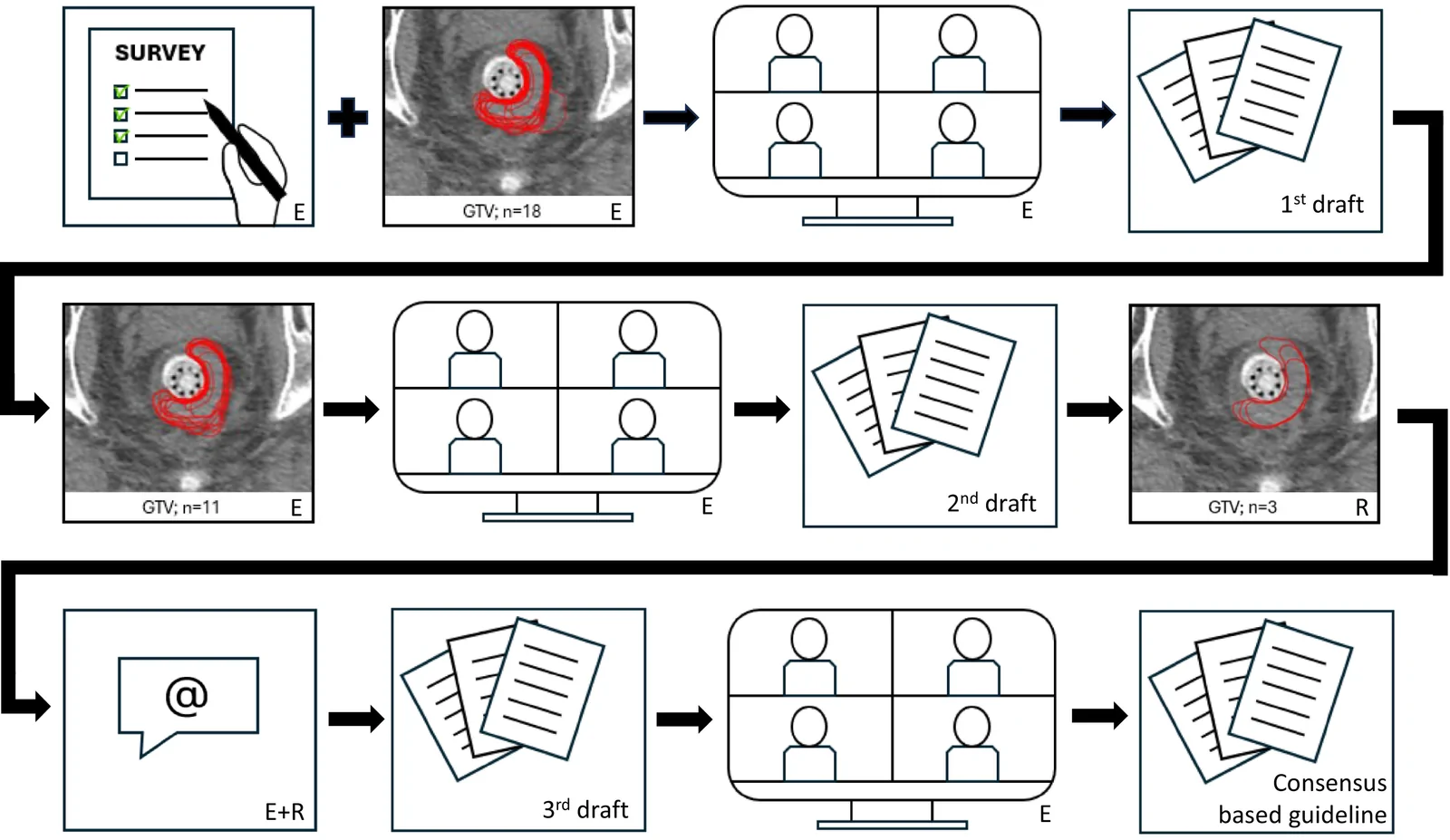
Schematic representation of the consensus process. E, expert group; R, review group.

*Step 1: Selection of an expert group.*

In August 2023, all GI GEC working group members, as well as additional experts proposed by them, were invited to participate in the project (“expert group”).

*Step 2*: *Survey.*

In September 2023, expert group members were invited to complete a survey regarding their centre's current practices in rectal EHB. Questions focused on experience with this technique, treatment intent, treatment protocols, target volume definition and OAR delineation.

*Step 3: First delineation round.*

Three rectal EHB cases were selected from different centres, varying in imaging modality (MRI vs. CT) and lymph node status (node-positive vs. node-negative). Each case included a description with physical examination results, diagnostic imaging, endoscopy reports, and a planning CT or MRI, all shared on the ESTRO FALCON platform. Expert group members were invited to delineate the target volumes and OAR for these cases according to their current practice. Table 1 summarises case characteristics. More details on the case information can be found in the supplementary material (S1).

**Table 1:** Summary of characteristics of delineation cases. CRT, chemoradiotherapy; MRF, mesorectal fascia; EHB, endoluminal high dose rate brachytherapy; EBRT, external beam radiotherapy.

	Staging	Previous treatment	Treatment	Delineation modality
Case 1	Local recurrence	CRT 4.5 years earlier (excellent response), no surgery; 2nd-line chemotherapy	EHB alone	CT
Case 2	cT3bN1M0, MRF-	None	EHB alone	MRI
Case 3	cT3bN1M0, MRF-	None	5x5Gy EBRT + EHB	MRI

*Step 4*: *First expert group meeting + draft recommendations.*

In April 2024, the first online expert group meeting was organised. The aim of this meeting was to develop an initial draft of the recommendations. During the meeting, the results of the survey were presented to the expert group members, and observed variations in delineated target volumes and OAR were discussed. Based on these discussions, statements were formulated regarding structure definitions and the prerequisites for adequate delineation. Statements on which all experts agreed were incorporated into the initial draft of the recommendations. Topics for which consensus could not be reached were discussed further in the subsequent expert group meeting.

*Step 5*: *Redelineation by expert group.*

The initial draft recommendations were provided to the expert group members. Based on the statements formulated regarding prerequisites for adequate delineation, additional information was incorporated into the patient cases on the ESTRO FALCON platform, such as clinical drawings to guide delineation of target volumes. Expert group members were invited to repeat the delineation of the target volumes and OARs for the three cases using the initial draft of the recommendations. Written feedback on the draft was obtained.

*Step 6*: *Second expert group meeting + second draft recommendations.*

A second online expert group meeting was organised in June 2024. Results from the second delineation round and feedback on the first draft recommendations were discussed. Topics on which no consensus had been reached during the first meeting were revisited. To facilitate the discussion, relevant literature and expert input on these topics were presented. If all experts agreed on a proposed definition or adjustment to an existing definition, the outcome was incorporated into the second draft recommendations.

*Step 7: Review of the second draft recommendations by the expert group and a review group + third draft recommendations.*

Expert group members were invited to review the second draft recommendations. A ‘review group’ was formed consisting of radiation oncologists (ROs) with experience in rectal EHB who were not involved in the development of the recommendations and had not participated in the expert group meetings. This ‘review group’ was asked to delineate the three patient cases on the ESTRO FALCON platform using the draft recommendations and to provide feedback. Based on the feedback from both the expert and review group, third draft recommendations were provided to the expert group, including systematic replies to all proposed revisions.

*Step 8: Third and final consensus meeting + final consensus recommendations.*

In September 2024, a final online expert group meeting was organised to reach consensus on the final points of discussion as a result of the received feedback. The third draft recommendations were adjusted to form the final consensus recommendations in November 2024.

## 3 Results

The expert group consisted of 30 experts in the field of rectal brachytherapy, general brachytherapy or gastrointestinal radiology. This included 26 ROs, two clinical physicists, a radiation technologist (RTT) and a radiologist from 21 centres in Austria, France, Germany, Italy, the Netherlands, Poland, the United Kingdom, Canada and India. The review group consisted of four ROs from Austria, France, Germany and the Netherlands. One of the ROs in the review group was initially part of the expert group, but only participated in the first delineation round. As a result, she was not involved in the development of the draft recommendations and was therefore considered independent.

The survey was completed by 16 experts, of whom 15 ROs and 1 RTT, from 12 centres in eight countries (supplementary material S2). Years of experience in rectal EHB ranged from 1 to 15 (median 7). Eight experts indicated that less than 5 patients were treated annually with rectal EHB at their centre, while only five experts indicated that more than 10 patients were treated annually. Two of these five specified treating around 50 and 70 patients per year.

Most centres provide both palliative and curative brachytherapy. Imaging modality at time of diagnosis always includes pelvic MRI, and in most centres also CT-scan and flexible endoscopy. Some centres use PET-CT as an optional imaging modality, e.g. to differentiate between scar tissue and local relapse or in cases of indeterminate nodes. Most patients receive EBRT and/or chemotherapy before brachytherapy. In curative settings, brachytherapy is often combined with long-course chemoradiotherapy. In palliative settings, most patients receive short-course EBRT in combination with brachytherapy.

In most centres, diagnostic MRI is repeated after neoadjuvant treatment, before EHB, sometimes with the applicator in situ to assist planning. Ten centres reported they insert markers around the tumour to guide EHB delivery. The two centres which do not place markers use planning MRI rather than CT. Most centres use CT for EHB planning; only one centre uses X-ray and plans to shortly move to CT planning.

Most centres plan and treat on the first fraction and adapt their plan on every subsequent fraction. In most cases, gross tumour volume (GTV) is defined as tumour on MRI at the time of EHB, but other modalities such as tumour visible on endoscopy or palpable at digital rectal examination (DRE) are taken into account during delineation. In most centres clinical target volume (CTV) is delineated as identical to GTV with no margin added to form the planning target volume (PTV). In general, lymph nodes are not considered as a target volume for EHB; positive lymph nodes can be treated with EBRT boost. However, some centres include nearby lymph nodes in a palliative setting. Heterogeneity was seen in the answers of individuals working within the same centres.

In the first delineation round, the first case was delineated by 19 ROs, the second one by 16 ROs and the third by 15 ROs. As all experts initially delineated according to their own insight and/or institutional guidelines, there was variation in the naming of structures, e.g. GTV, high risk CTV (HR-CTV), intermediate risk CTV (IR-CTV), low risk CTV (LR-CTV), CTV, PTV and various combinations. It was noted that when delineating on CT, experts most often defined a single target volume, whereas MRI-based delineation more commonly involved the use of margins to create multiple target volumes. In the second round, the three different cases were delineated by 11, 10 and 10 ROs, respectively. In total the first, second and third expert group meetings were attended by 15, 14 and 14 RO's respectively. During the first expert group meeting, it was decided to focus the recommendations on curative treatment, given that palliative treatment often involves different clinical considerations.

Consensus was reached on 3 areas: preconditions for adequate delineation, target volume definitions and OAR delineation.

Certain preconditions are needed to accurately determine the target volume. Endoscopy and DRE should be performed prior to the first applicator placement. If a (diagnostic) endoscopy has been performed more than one week before EHB, a new endoscopy is advised. This is essential when the patient has been treated in the intervening period with EBRT, as the majority of tumours will have shown significant regression [18]. A new endoscopy before each applicator placement is strongly recommended as the tumour may change between EHB fractions as well. In settings where repeated endoscopy is not available, a DRE alone, when performed by an experienced clinician, may serve as a reasonable alternative, if the tumour can adequately be assessed by DRE.

If available, it is recommended to perform a rigid rectoscopy rather than a flexible endoscopy. It can provide a better orientation of the tumour, as the degree of rotation is difficult to estimate for a flexible endoscope. If rigid rectoscopy is not available, it can be helpful to use the “water level” for a better orientation during flexible endoscopy (Fig. 2).

**Fig. 2:**
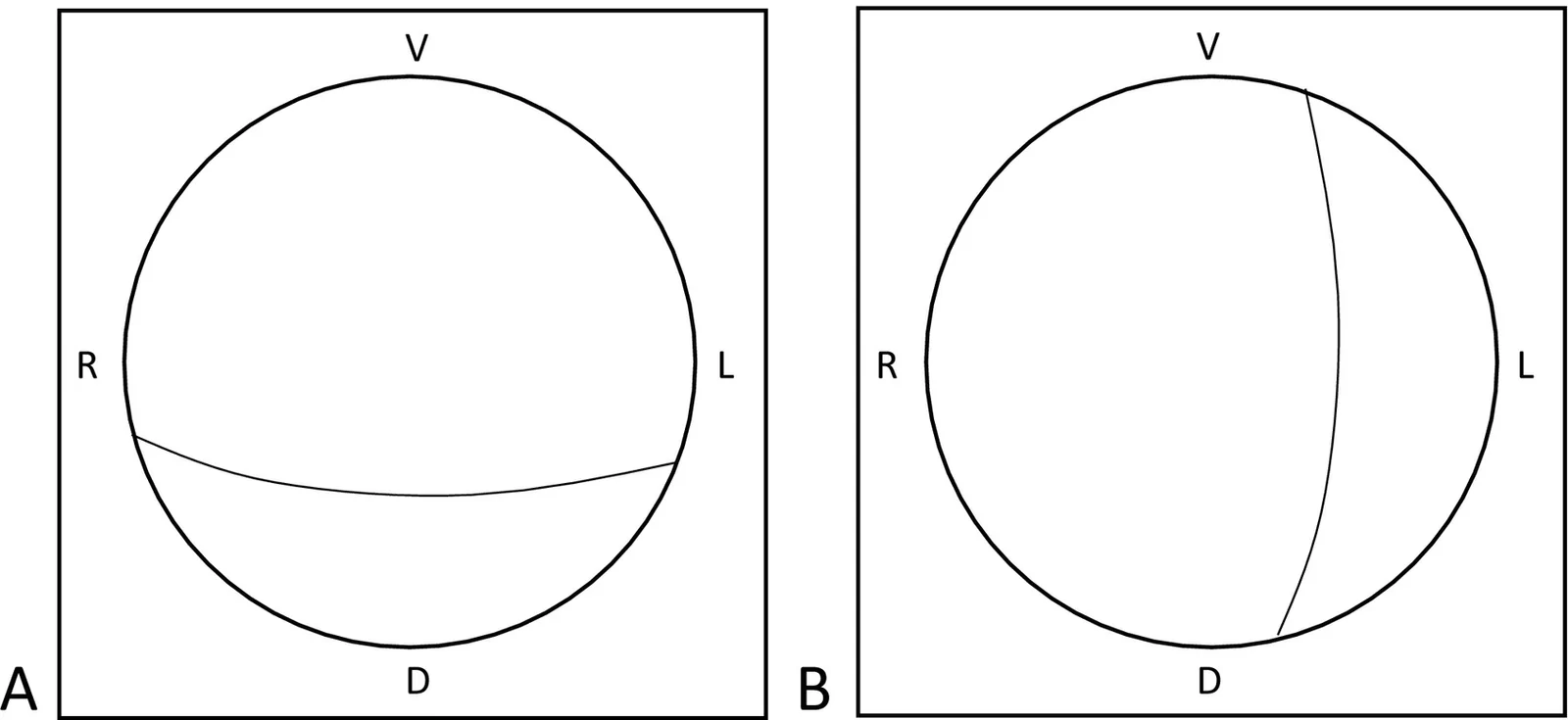
The water level can be helpful for orientation, when the position of the patient is known. Shown in clockwise position, with 12 o'clock corresponds to the ventral side of the rectum. A. a patient lying in lithotomy position, with the water dorsal in the rectum due to gravity. B. a patient lying in the left lateral position. V, ventral; L, left; D, dorsal; R, right.

The endoscopy report should contain at least:

• Tumour position, preferably using the clock face (with the 12 o'clock position being the ventral side of the patient), showing the lateral borders of the tumour
• Distance from anal verge to caudal tumour border. It can be measured by reading the length markings on the exterior of the endoscope (counting down from the tip)
• Tumour length in craniocaudal direction (to establish the cranial tumour border)
• Number and length of markers and location in relation to the tumour (if used)
• Position of the patient during the endoscopy

Endoscopy pictures and/or clinical drawings with precise location of the tumour and markers, including orientation of the picture are recommended. A template can be found in the supplementary material (S3); however, each centre is free to use its own template. It is recommended that the RO is present during endoscopic evaluation and placement of markers.

The use of radiopaque fiducial markers, e.g. endoscopic clips, enables accurate tumour localisation when CT or 2D imaging is used and is therefore recommended in those cases. MRI compatible markers can be of added value in case of MR-based EHB, particularly for small residual lesions where imaging interpretation can be difficult, although their use is not essential. Since healthy rectal mucosa can be stretched, it is recommended to place the markers as close to the tumour as possible. The estimated distance from each of the markers to the tumour border should be reported, which in case of clips is defined as the distance from the tumour edge to the grip end of the clip on the rectal wall. Cranially placed markers should demarcate the cranial border and caudally placed markers the caudal border of the tumour. It can be helpful to place markers to the lateral borders of the tumour, to help guide clockwise position (these can be the same as the cranial or caudal markers); although the endoscopy report and/or DRE are often sufficient. In general, it is recommended to place up to 4 markers, when demarcating the cranial and caudal border (e.g. 2 cranial and 2 caudal), as there is always a chance of losing markers over time. When treating a proximal tumour with the use of 2D imaging for position verification of the applicator, it is recommended to place 3 markers rather than 4 (e.g. 2 cranially and 1 caudally), as there is a chance that the rectosigmoid – and thus the cranial marker – is bending downwards, caudally of the caudal marker, if the applicator has not been placed proximal enough (Fig. 3). It is recommended to endoscopically verify the marker positions just before each EHB procedure, but at least within one week of each EHB procedure due to the risk of losing them over time and/or changes in tumour volumes.

**Fig. 3:**
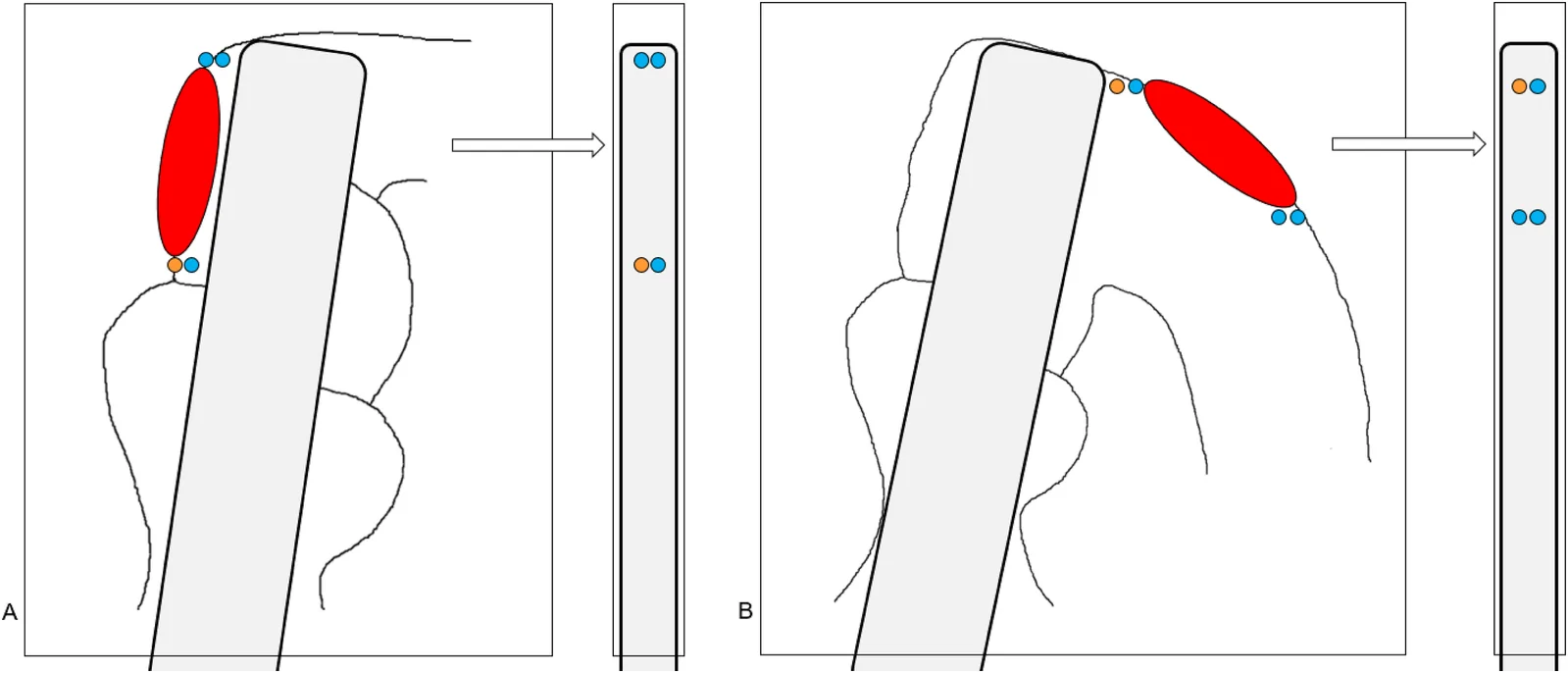
Asymmetric marker configuration for proximal tumours. A. The applicator is placed correctly, verified on 2D imaging using an asymmetric marker configuration (blue markers). B. The rectosigmoid is falling downwards here. If a symmetric four-marker configuration is used (blue and orange markers), 2D imaging may erroneously indicate correct applicator placement. (For interpretation of the references to colour in this figure legend, the reader is referred to the web version of this article.)

When using clips, there are several additional considerations to take into account. As it is difficult to localise the grip end of a clip, the delineation uncertainty can be the entire length of the clip. Therefore, it is advised to use the smallest available clips, with good visibility characteristics on the medical imaging modality used (Fig. 4). It is not recommended to place the clips directly on the tumour as they can dislodge more easily. Moreover, caudally placed clips should be avoided if it would result in placement within the anal canal, as this may cause significant patient discomfort.

**Fig. 4:**
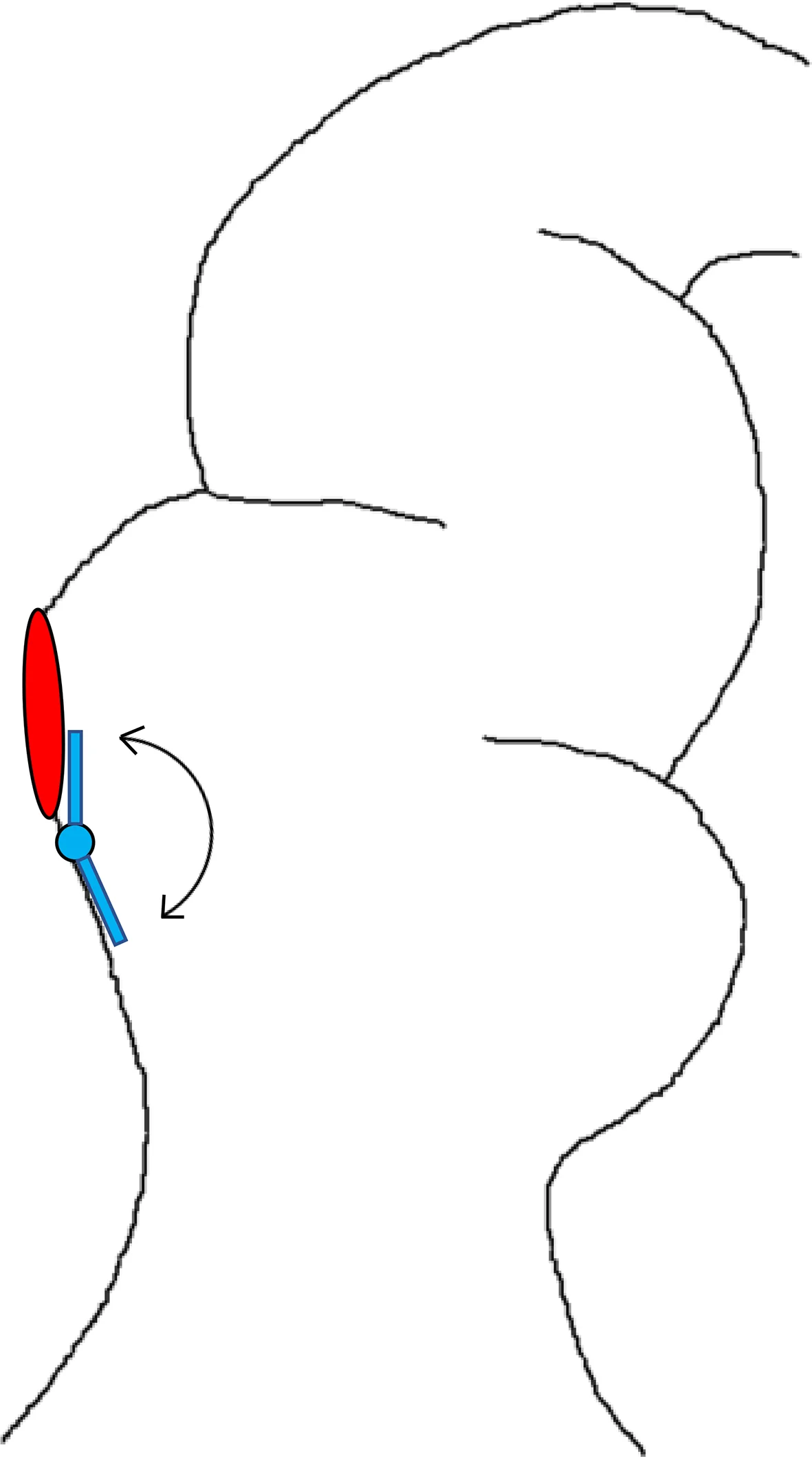
Two possible positions of the clip. The circle visualises the grip end; the clip can rotate around its grip end.

Before imaging, an enema is strongly recommended to reduce variation in faecal load, making it easier to compare scans. A diagnostic pre-EHB MRI (T2 and DWI (Diffusion-weighted imaging)) should be available, especially when CT is used for planning. Preferably, the MRI should be less than 2 weeks old at the time of EHB [18]. When EHB is delivered following EBRT, the MRI is performed post EBRT. If available, rectal gel filling with a defined volume can be used to improve visibility of the thickened mucosa. However, the latter needs to be done with caution, as it can compress the tumour against the mesorectal fascia (MRF), leading to overestimation of MRF involvement and hampering of lymph node evaluation. It may also cause T2 shine-through effects on DWI, which can look like diffusion restriction. Limit the volume of gel to less than 60 cc to reduce the risk of compression of the tumour [19], [20].

Target delineation and treatment planning is performed on imaging with the applicator in situ. MRI is preferred, but CT imaging with markers is an acceptable alternative. When MRI is used, an MRI-compatible applicator is strongly recommended for safety and to avoid artefacts. As a minimum, images should be obtained in two planes (or 3D volumetric MRI).

On MRI, macroscopic tumour is seen as a hyperintense zone on T2-weighted images, in combination with a high signal on DWI. However, in cases of any heterogeneous or isointense signal on T2 and restricted diffusion on DWI, macroscopic disease cannot be excluded. T2-weighted imaging is preferred and should be interpreted in line with endoscopy findings to ensure macroscopic disease is covered. DWI is a helpful addition in terms of distinguishing scar tissue from healthy mucosa; however, its added value depends on MRI compatibility and the associated risk of applicator-related artefacts. We recommend involving an experienced radiologist if there is any doubt on GTV delineation.

When delineating on a treatment planning CT, high resolution CT is essential. Axial images are obtained with reformatting in sagittal and coronal planes. Slice thickness should be 3 mm or less. There is no evidence for the addition of IV/oral contrast. If a balloon applicator is used, use of liquid contrast (instead of water) may aid identification [21]. In case of CT-based delineation, fusion of the pre-EHB MR with the planning CT can be considered. However, uncertainties due to applicator placement, tumour response, patient position, and rectal filling should be taken into account.

When delineating, always take into account the location of markers (e.g. their clockwise position and distance from tumour borders). In the case of clips, length is also relevant, particularly when the grip end cannot be localized on imaging. Note that clips, due to placement of the grip end outside the tumour, are not automatically included in the GTV for EHB.

Recommended structure naming follows ICRU recommendations and AAPM TG-263 [22], [23]. A summary can be found in Table 2 A. Fig. 5 shows an example of the delineated structures. Endoscopy findings should lead in delineation of the GTV primary at the time of EHB (GTVp_BT), since historically the standard of rectal EHB is treating the tumour volume as seen endoscopically. This approach has been clinically validated using CXB, demonstrating very good results regarding 3 year local control (97% for tumours smaller than 3 cm, 80% for all tumours treated with CXB in the OPERA study) [8]. Information gathered from other diagnostic procedures such as radiological imaging and clinical examination should also be incorporated. For example, in the case of a discrepancy between endoscopic and radiological assessment of residual tumour – e.g. no obvious endoscopic residual tumour, but diffusion restriction on MRI – the GTVp_BT volume may need to be adapted. Extramural vascular invasion (EMVI) or tumour deposits that are in continuity with the tumour should be included in the GTVp_BT. Lymph nodes and non-continuous EMVI or tumour deposits should not be included. When replanning over different fractions, only the residual tumour should be delineated as GTVp_BT, because of centripetal shrinkage [24]. In such cases, the fraction number should be added to the structure name, e.g. GTVp_BT1.

**Table 2:** A: Summary of target volume definitions; EMVI, extramural vascular invasion; B: OAR contouring instructions. The descriptions of the Rectal_Wall, Anal_Canal, Colon_Sigmoid, Bladder, Bowel, Urethra, and PenileBulb have been adapted from the Global Harmonization Group organ at risk consensus contouring guideline [25]. Partially contoured structures are denoted with the tilde (~) suffix.

Target volume	Standardised naming	Definition	Notes
GTV primary at the time of EHB	GTVp_BT	Macroscopic primary tumour at the time of EHB	GTVp_BT includes tumour deposits and EMVI in continuity with the primary tumour.
GTVp_BT with expansion of 5 mm Evaluation structure	GTVp_BT_ev05	Volume that potentially contains relevant microscopic disease	GTVp_BT with a margin of 5 mm in all directions. The applicator, endorectal balloon(s), stool, rectal lumen and surrounding uninvolved OARs are excluded.
GTV node	GTVn_BT	All visible remnants of initially suspected lymph nodes on brachy planning imaging, within 4 cm of the macroscopic primary tumour volume.	GTVn_BT includes tumour deposits and EMVI that make **no** direct contact with the primary tumour.


	OAR	Definition / Anatomic location
Delineation for the purpose of standard reporting	Rectal_Wall_CL (Rectal _Wall_Contralateral)	Rectal wall at the level (in cranio-caudal direction) of GTVp_BT minus GTVp_BT (i.e. GTVp_BT should be excluded).
	Rectal_Wall	Outer contour of the rectum, including GTVp_BT, excluding applicator, stool and rectal lumen. Cranial border: the sigmoid take-off. Caudal border: the anorectal junction, where the perirectal fat can no longer be seen, corresponding with the insertion of the levator muscles and the puborectal sling.
	Anal_Canal	Outer contour of the outer sphincter, from the level of the anorectal junction to the anal verge; excluding applicator. The anal canal originates at the anorectal junction where the perirectal fat can no longer be seen, coinciding with the insertion of the levator muscles and the puborectalis sling; these structures are best visualised on coronal viewing planes. The anal canal continues caudally to the anal verge. The contour adheres closely to the outer boundary of the external anal wall, and includes sphincter muscles.
	Colon_Sigmoid	The sigmoid colon is an S-shaped structure that is mobile on its own mesentery. The cranial border is where the structure meets the descending colon and can be identified where the colon curves medially at the level of the left iliac crest. The caudal border is at the sigmoid take-off. The contour adheres closely to the outer boundary of the external wall and includes sigmoid bowel contents.
	Bladder	The bladder should be contoured in entirety from base to dome. The lateral extent is the outer bladder wall.
	Vagina	Outer contour of the vagina. The caudal border of the vagina can be determined by the ischial tuberosity or the lower border of the urethra.
Delineation for exploratory reporting	Bowel ~	The bowel encompasses the outer contour of small (duodenum, jejunum and ileum) and large bowel (caecum, ascending, transverse, descending, and sigmoid colon) structures in one contour, from the pylorus to the sigmoid take-off. It should only be delineated within 4 cm of the GTVp_BT.
	Sacrum	Whole sacrum, including coccyx.
	Urethra	The urethra extends from the internal urethral orifice at the bladder neck and continues caudally to the external urethral orifice. In females, the urethra is 4 cm in length. In males it is 17.5-20 cm in length. The urethra is not visible on CT and should be contoured on T2-weighted MRI where it is moderately hyperintense. Contour to include all muscle layers and use sagittal viewing planes to aid identification of the structure. Be aware that placement of a urinary catheter may distort the position and shape of the urethra.
	Prostate	The outer contour of the prostate, from the base to the apex; excluding vesicles.
	PenileBulb	The penile bulb is the portion of the bulbous spongiosum of the penis immediately caudal to the genito-urinary diaphragm. The structure is bright on T2 weighted MRI. On CT, the structure is posterior to the urethra and has a round shape. The structure is normally 9-10 mm in the cranial-caudal direction. The contour should not continue in to the shaft of the penis.

**Fig. 5:**
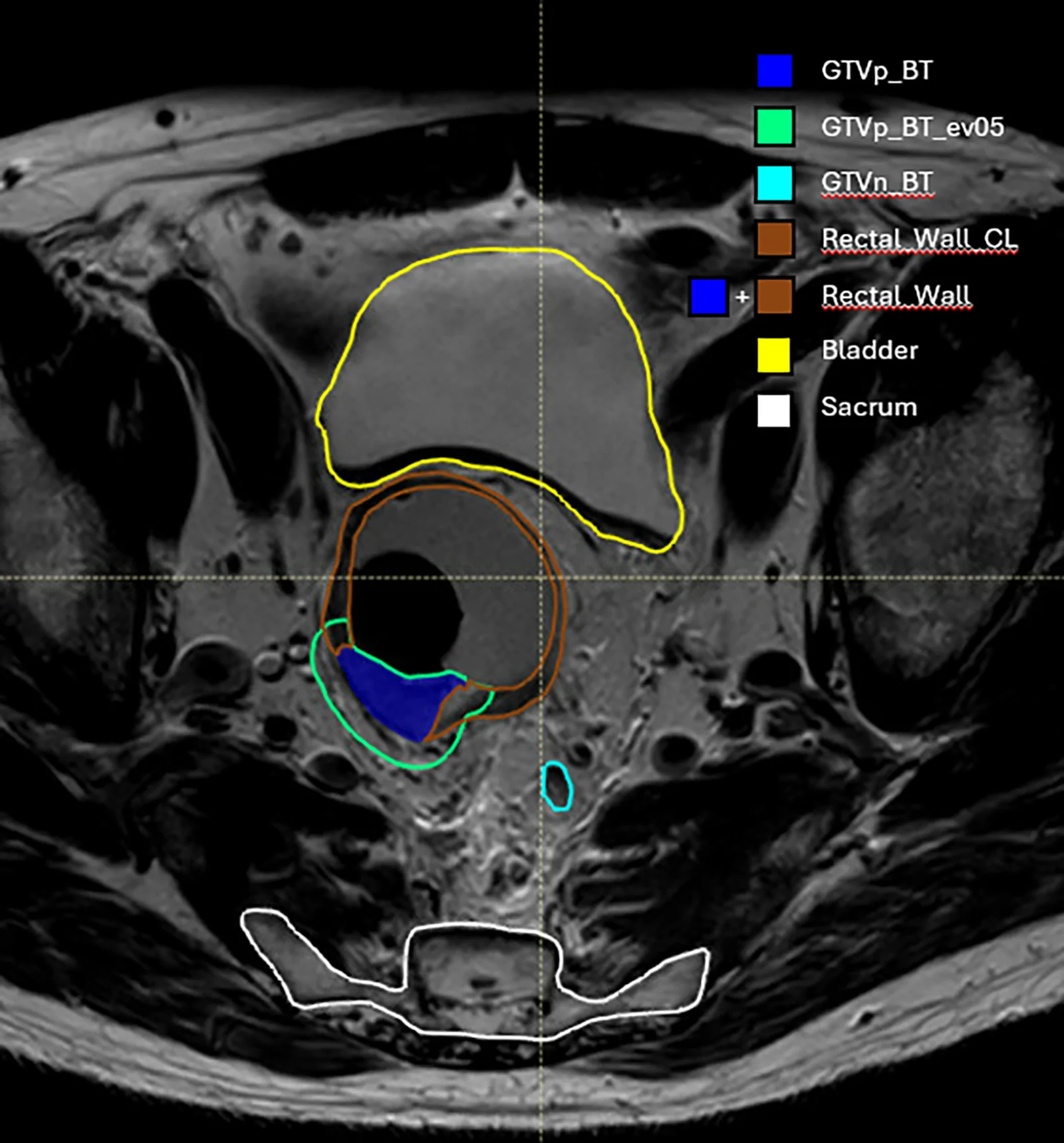
Example of delineation on an axial MRI image.

Based on evidence that a 5.5 mm margin from the GTVp_BT is required to cover microscopic tumour spread (MTS) in 95% of cases following neoadjuvant chemoradiation (NA-CRT) [24], we defined an evaluation structure GTVp_BT_ev05, corresponding to the GTVp_BT with 5 mm expansion. This volume is intended for dose evaluation purposes only, in line with TG-263 nomenclature [23]. It enables analysis of the dose delivered to potential microscopic tumour spread (MTS). The applicator, endorectal balloon(s), stool, rectal lumen and surrounding uninvolved OARs are excluded from the GTVp_BT_ev05. In case of replanning over the different fractions, the GTVp_BT_ev05 should change along with the changing GTVp_BT, as it is reasonable to assume that the shrinkage pattern remains the same [24].

The GTV node (GTVn_BT) contains all visible remnants of initially suspected lymph nodes on the EHB planning imaging within 4 cm of the macroscopic primary tumour volume. It includes lymph nodes, EMVI and tumour deposits that are not in continuity with the primary tumour. It should be noted that lymph nodes are generally treated with EBRT rather than EHB. However, reporting the dose received by the lymph nodes during EHB may aid evaluation of local control outcomes.

The selection of OARs (Table 2b) is based on toxicity reported in literature [15]. Based on discussions during the expert group meetings, several modifications were made. The OARs were categorised into two levels: those for the purpose of standard reporting, which should be delineated by all institutions, and those for exploratory reporting, which should be delineated by research institutions. The definitions have been adopted from the AAPM TG-263 and the Global Harmonization Group organ at risk consensus contouring guideline [23], [25]. Where applicable, the rectosigmoid junction was replaced by the sigmoid take-off, due to its easier localisation [25].

OARs should be delineated in their entirety, except for the bowel; only loops within 4 cm of the GTVp_BT should be contoured. However, complete delineation may not always be feasible, particularly on CT imaging, due to insufficient anatomical visibility. In cases with substantial doubt, we recommend delineating only the visible part of the OAR, adding the tilde suffix (~) [25], as mislocalisation may compromise the accuracy and value of the contour. For example, delineation of the urethra, penile bulb, and vagina may not be feasible for this reason. Ensure that anatomical continuous structures as the sigmoid colon, rectum and anal canal are also represented as contiguous in the planning structure (i.e., without any gaps between them). Depending on the planning system used this might mean that structures are delineated with or without one overlapping slide.

## 4 Discussion

The GI GEC working group of the GEC-ESTRO group initiated the rectal EHB project to improve the quality of rectal EHB throughout Europe and beyond. The successful implementation of the GEC-ESTRO recommendations for cervical cancer [26], [27], which resulted in standardised treatment and improved clinical outcomes, illustrates the potential impact of such initiatives. Following the publication of the consensus recommendations for CXB in 2022 [11], the present work focuses on the standardisation of target volume and OAR definitions in rectal EHB. This will form the basis for recommendations on dose reporting.

The survey conducted at the start of our project revealed a broad heterogeneity in treatment approaches, even within individual institutions. The intra-institutional variation likely stems from the absence of institutional guidelines, further underscoring the need for this project. In this project expert consensus was reached on preconditions for adequate delineation, target volume definitions for dose reporting, and OAR delineation.

To accommodate the diverse workflows across institutions, the recommendations include alternatives to the recommended standards, aiming to ensure recommendations that are relevant and applicable to heterogenous working arrangements. For instance, while MRI is the preferred modality for treatment planning, most institutions currently use planning CT. With the right preconditions, such as the use of markers, CT-based planning can be an acceptable alternative to MRI-based workflows.

While some procedural heterogeneity is acceptable, consistent and unambiguous nomenclature is essential for standardised dose reporting. In the currently available literature, identical terms, e.g. CTV, are often used to describe anatomically different volumes [28], leading to confusion. This became evident during the expert group meetings, where the term CTV frequently led to misunderstandings. Some experts suggested adopting the terminology used in cervical brachytherapy [26], to keep the analogy with cervical brachytherapy. However, due to the subsequent publication of the TG-263 guidelines [23], by consensus it was decided to include the macroscopic primary tumour at the time of EHB (GTVp_BT) as the primary tumour related dose reporting structure, as it was considered the most unambiguous definition. The selection of this dose reporting structure is in line with common clinical practice, as the macroscopic primary tumour as seen during pre-EHB endoscopy is often considered the main target volume for dose prescription. The addition of “BT” clarifies that the volumes refer to those delineated at the time of EHB rather than the original tumour volumes.

Nevertheless, based on the meta-analysis of Verrijssen et al. [24], it may be of clinical relevance to include a margin to cover potential MTS. This meta-analysis has some limitations. First, the direction of MTS could not be determined, as most studies primarily focused on distal extension parallel to the bowel wall. In cases without extramural extension at diagnosis, perpendicular MTS is likely minimal, and a smaller margin may suffice. The ongoing prospective DETECT trial (NCT04927897) aims to provide more detailed information on MTS in all directions by analysing resection specimens after neoadjuvant (chemo)radiotherapy. Additionally, the meta-analysis did not include tumours without NA-CRT, although some evidence suggests that the same margin would suffice in patients not receiving NA-CRT [29]. Since the clinical relevance of a margin has not yet been proven, we decided to introduce an evaluation structure (GTVp_BT_ev05) rather than to define a CTV. Based on future reported data, we aim to conduct analyses to assess the clinical relevance of the dose to the GTVp_BT_ev05. Moreover, we aim to assess the relevance of the structure in different clinical scenarios, e.g. in tumour with/without extramural extension, and in scenarios with/without NA-CRT.

These consensus recommendations do not define target volumes specifically for dose prescription, as the sole aim of the current project is to provide a set of structures for standardised dose reporting. Dose reporting and prescription are two different entities: dose reporting describes the dose actually delivered to a defined volume, whereas dose prescription refers to the intended dose to a target volume, which may or may not be achieved within a certain clinical context. Due to the absence of standardised dose reporting, there is insufficient data to provide evidence-based recommendations on what the optimal treatment volumes and associated prescription doses should be, e.g. should a margin to cover potential microscopic tumour spread be included? Accordingly, for now, clinical dose prescription practices should not be adapted based on the current recommendations, as a lack of variation may hamper the possibility to identify dose–response relationships. Likewise, institutions may define their own CTVs and, if applicable, PTVs. When institutions define their own volumes, nomenclature consistent with the current recommendations is advised, e.g. PTVp_BT.

At this stage OARs are delineated in their entirety wherever feasible. Only once the specific dose parameters to be reported have been evaluated in the next phase of the project it will become clear whether partial delineations are feasible. The definitions of the rectal volumes were the subject of the most extensive discussion. It was decided to include the tumour volume within the total rectal OAR contour. This decision was based on the assumption that irradiation of a larger tumour may result in increased toxicity compared to irradiation of a smaller tumour, assuming the same dose to the healthy rectal tissue, as there is a reduced amount of functional rectal tissue remaining after irradiation of a larger tumour. Moreover, the tumour volume can be subtracted from the delineated structure to allow for more accurate analysis if necessary. The contralateral bowel mucosa should be delineated separately, based on the hypothesis that a relationship may exist between the dose specifically to this volume and subsequent late bowel strictures. As rectal toxicity, including fibrosis, is known to be dose-dependent [30], a higher dose to the contralateral bowel wall may contribute to extensive circumferential fibrosis at the level of the tumour, potentially resulting in bowel strictures.

A major strength of this project is that the expert group comprised of recognised experts in the field, from diverse institutions, who also reflect the intended target users of the recommendations. However, there are several potential limitations. First of all, a systematic review was not performed at the outset. The recommendation is therefore primarily based on expert consensus, although supporting evidence from the literature was cited where appropriate and available. While not all experts were involved in every phase of the project, all members reviewed the final version of the consensus recommendations and were given the opportunity to provide feedback. Secondly, the lack of anonymity during the consensus meetings may be considered a limitation, as anonymity is a key issue in a Delphi approach; anonymity could encourage honesty and help reduce the influence of dominant individuals – a phenomenon known as the “halo effect” [31]. However, implementing anonymity was not feasible within the context of in-person discussions. A third potential limitation is the delineation of only three clinical cases, of which two were palliative, as a basis for discussions. We opted for a balance between workload and ensuring sufficient case diversity. The selected cases were specifically selected to cover diverse clinical contexts, including curative vs. palliative treatment, planning on MRI vs. CT, and N+ vs. N0 staged tumours. The decision to focus the recommendations solely on curative treatment was made during the project. Nevertheless, the three cases facilitated comprehensive discussion and addressed the most relevant diversities in potential clinical scenarios. Finally, some survey questions may not have been formulated with sufficient clarity. Although the survey was intended to address rectal EHB only, comments indicated that at least some experts also considered CXB in their responses. Since the primary purpose was to obtain a general overview of current practices, and the survey was followed by extensive discussions, we do not believe this issue significantly influenced the development of these recommendations.

These delineation recommendations establish a solid foundation for the next phase of the project, in which we will define the recommended dose reporting parameters. Together, the two sets of recommendations aim to standardise dose reporting to its full extent. The second set will also provide guidance on dose prescription and treatment planning, as well as on patient selection, treatment preparation, and follow-up.

The consensus recommendations are expected to be relatively easy to implement across different centres, since in addition to the recommended standard, alternative options are often provided. The involvement of experts from multiple countries may also facilitate implementation within respective countries. Future ESTRO educational activities, such as FALCON workshops and contouring atlases, may further support implementation. Broad adoption of a standardised delineation framework may not only improve consistency in clinical practice, but also facilitate multicentre comparisons, support the development and validation of TCP and NTCP models, and provide a foundation for future AI-based auto-contouring tools. Validation of these consensus-based recommendations and their relationship with clinical outcomes will require prospective data collection, ideally through international registries and collaborative studies. Components of these recommendations may be revised based on insights gained during the development of the second set of consensus recommendations, or if clinical experience or findings from other studies indicate the need for revision.

## 5 Conclusion

The GI GEC working group of GEC-ESTRO have developed consensus recommendations for target volume definition and OAR delineation in EHB for rectal cancer. These recommendations will be implemented in clinical practice and used to develop recommendations for dose reporting.

## Disclaimer

ESTRO guidelines present scientific and medical opinions for educational and informational purposes only. Commercial use of any content in the guidelines without the prior written consent of ESTRO is strictly prohibited. The guidelines are not, and nor are they intended or implied to be, a substitute for professional medical advice or medical care. The advice of a medical professional should always be sought prior to commencing any form of medical treatment. Under no circumstances will ESTRO be held liable for any decision or action taken as a result of reliance on the content of the guidelines. ESTRO and all of its staff, agents and members disclaim any and all warranties and representations (implied or otherwise) with regards to the information contained on the guidelines. Each guideline is based on information available at the time the corresponding writing panel conducted its research and discussions on the topic. Guidelines should neither be deemed inclusive of all proper methods of care or factors influencing the decision making, nor exclusive of other methods reasonably directed to obtaining the same results. There may be new developments since publication that are not reflected in any one guideline and that may, over time, form the basis for ESTRO to revisit and update the guideline.

## Declaration of competing interest

The authors declare that they have no known competing financial interests or personal relationships that could have appeared to influence the work reported in this paper.

## Appendix A Supplementary data

Case information.

Survey results.

Endoscopy template.
