# Supplementary material for: ESTRO consensus recommendations: target volume and organ at risk delineation in rectal endoluminal high dose rate brachytherapy
Source: Clin Transl Radiat Oncol. 2026 Oct 31;62:101341. doi: 10.1016/j.ctro.2026.101341 (PMC13769233; doi:10.1016/j.ctro.2026.101341)
Supplement: Supplementary file 1 [file mmc3.pdf]

Endoscopy

Patient ....
Date ....

☐ At diagnosis
☐ Pre-radiotherapy
☐ At brachytherapy fr ....

Position of patient ....

☐ Flexible / ☐ Rigid rectoscopy

Distance from anal verge = ... cm
Distance from top of sphincter = ... cm
Tumor length = ... cm
Tumor width = ... cm
O'clock position = ... h to .... h
(Estimated) tumor thickness = ... cm

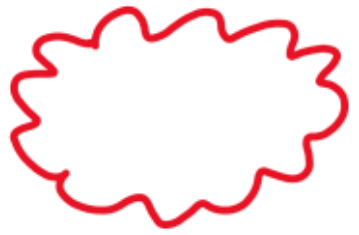

Polypoid tumor

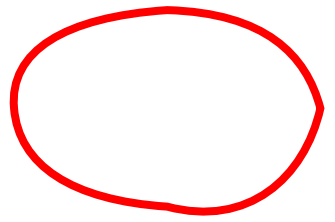

Flat tumor

Markers (mark with x)	Number	Location
Proximal		....
Distal		....

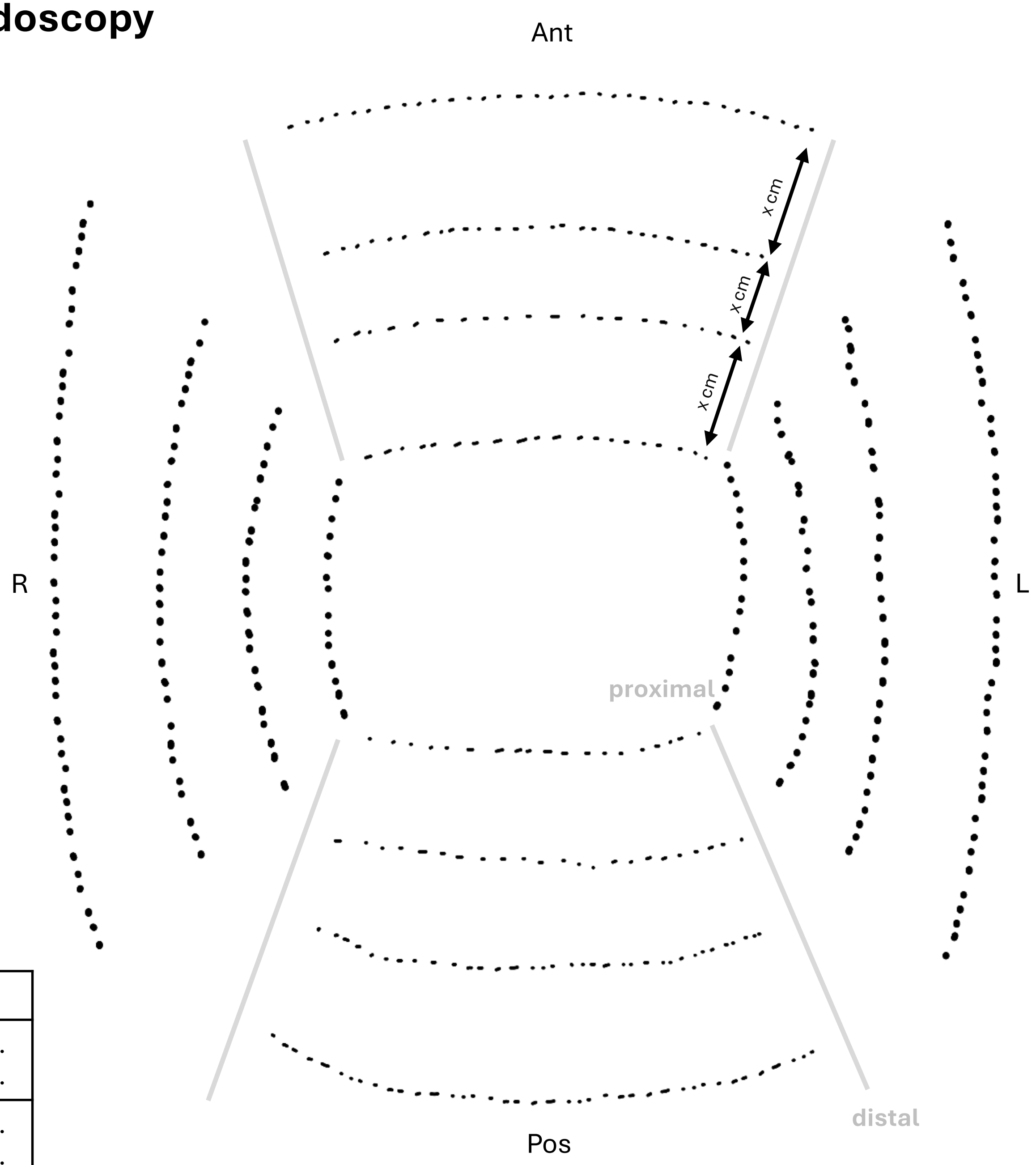

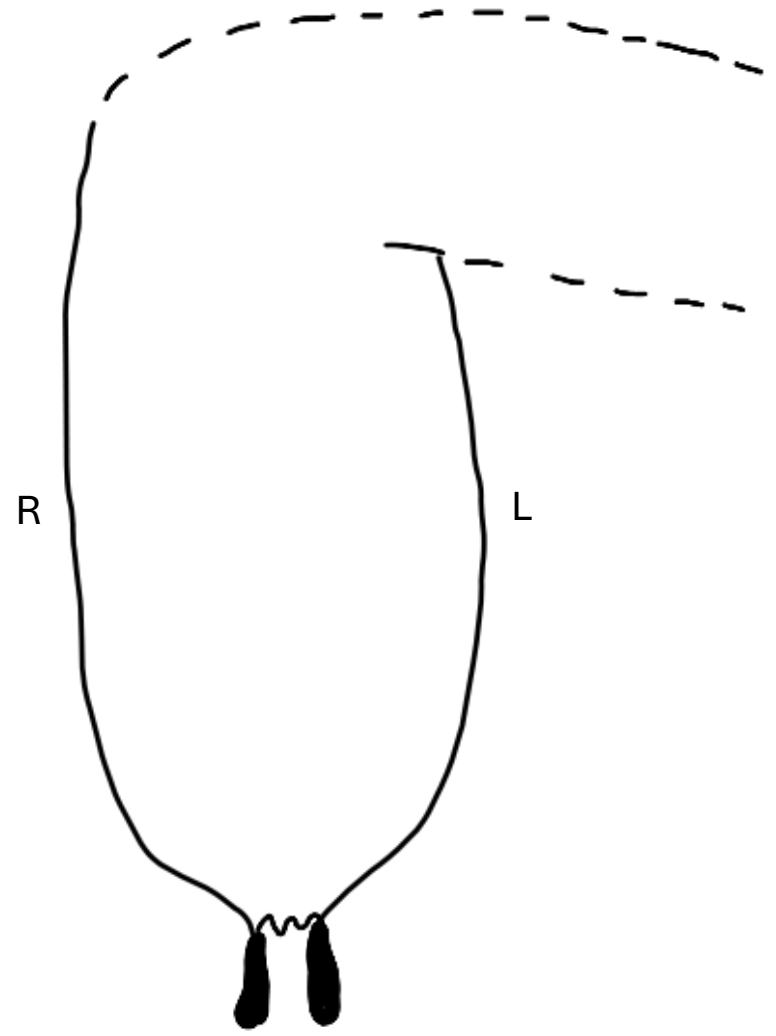

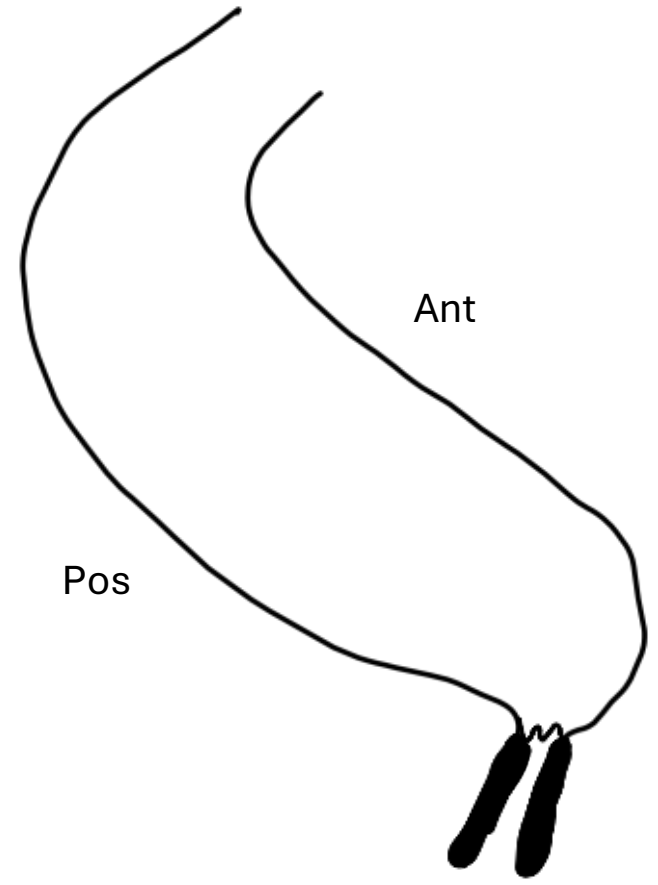

x = ... cm
