# Supplementary material for: ESTRO consensus recommendations: target volume and organ at risk delineation in rectal endoluminal high dose rate brachytherapy
Source: Clin Transl Radiat Oncol. 2026 Oct 31;62:101341. doi: 10.1016/j.ctro.2026.101341 (PMC13769233; doi:10.1016/j.ctro.2026.101341)
Supplement: Supplementary file 2 [file mmc1.docx]

**Clinical information patient 1**:

A 62-year-old man, medically inoperable. Received chemoradiotherapy for low rectal cancer 4.5 years ago (45 Gy -1,8Gy / fraction), excellent response. Now relapsed after second-line chemotherapy, symptomatic.

*MRI:* Recurrence. Tumour 12-8 o’clock in a clockwise direction. Extramural infiltration

from 3-6 o’clock. Intermediate signal soft tissue reaching outside rectal wall on the

left hand side reaching left levator. No Mesorectal LN.

*Biopsy*: Confirmed moderately differentiated adenocarcinoma recurrence of the

Rectum

*Rigid rectoscopy*: Malignant looking mass 0-6cm from dentate (so about 3-9cm). 2

clips above (placed at proximal tumour border at 12 and 8 o’clock), 1 clip below

(placed at distal tumor border at 8 o’clock)

*PET-CT*: Avid lung nodules and recurrent rectal mass

**Diagnosis:** local recurrence

**Treatment:** rectal HDR brachytherapy

*Available imaging during delineation on planning CT*: MRI at the moment of recurrence

**Clinical information patient 2:**

A 73 year-old-man. Rectal tumor discovered at colonoscopy indicated by a positive FOBT in the population screening programme. Complaints of blood loss.

**Colonoscopy before treatment:** At 11 cm from the anorectal verge a lesion, malignancy suspected. Lesion with stenosis, half of the circumference (from 4-10h) , length 3 cm, coecum reached, no other lesions.

*Biopsy*: gr II adenocarcinoma

*MRI before treatment*: 10 cm from the anorectal verge a tumor, 2 cm. Between 7 and 9 o’clock invasion through the musc propria, 3 mm extramural invasion. Mesorectal fascia free with a margin of 7 mm. No EMVI. Three suspicious mesorectal lymph nodes, 5,6 and 7 mm resp.

Classification T3bN1M0, MRF – EMVI –

*Endorectal ultrasound before treatment*: Tumor visualised at 15 cm from anal verge, 50% circumference. Tumor length 4-5 cm.

*MRI at first brachytherapy fraction*: Applicator in situ, stretch of rectum. Tumor position dorsal instead of dorsolateral on previous scan. Diameter of tumor 3 cm.

**Diagnosis**: rectal cancer T3bN1M0 MRF- EMVI-.

**Treatment:** palliative HDR brachytherapy (no EBRT)

*Available imaging during delineation on planning MRI*: MRI 1 month before treatment

**Clinical information patient 3:**

A 81-year-old man. Presented with haematochezia, urgency, and sometimes rectal incontinence for several weeks before diagnosis.

*Colonoscopy at diagnosis*: At 8 cm from the anorectal verge a lesion, malignancy suspected. Lesion with partial stenosis.

*Biopsy*: gr II adenocarcinoma

*MRI before treatment*: 5.2 x 4.4 x 4 cm mass in the rectum approx. 6 cm from the anorectal junction, with small streaky extensions into the mesorectal fascia as well as individual enlarged perirectal nodes up to 5mm. MR tomography therefore shows a T3b N1 tumour stage

*Proctoscopy one week before BT*: Tumor visualised at 6 cm, from 5 to 8h. Placement of four markers. The first marker rather far proximal from tumor at 8h. The second one at 1 cm proximal from tumor at 5h. The third and fourth both a 1 cm distal from the tumor.

*MRI at first BT*: Good response

**Diagnosis:** rectal cancer T3bN1M0

**Treatment:** Short course radiotherapy (25Gy in 5 fractions) to the pelvis with a sequential brachytherapy boost to the tumour with another fraction of 5 Gy (total dose at the tumour: 30 Gy)

*Available imaging during delineation on planning MRI*: MRI before EBRT
