# Supplementary material for: ESTRO consensus recommendations: target volume and organ at risk delineation in rectal endoluminal high dose rate brachytherapy
Source: Clin Transl Radiat Oncol. 2026 Oct 31;62:101341. doi: 10.1016/j.ctro.2026.101341 (PMC13769233; doi:10.1016/j.ctro.2026.101341)
Supplement: Supplementary file 3 [file mmc2.docx]

1. How many years of experience do you have with rectal brachytherapy?

**Answer**

4

8

6-7

7

8

10

6

15

15

4

6

1

10

3

4

12

2. How many patients are treated annually with rectal brachytherapy at your institution?

| **Answer** | **Comments** |
| --- | --- |
| >10 | Around 20 |
| <5 | we only use this option for frail rectal cancer patients after external beam irradiation, preferably in the context of a clinical study. Our last study closed prematurely due to accrual problems, since then we did not treat any new patients. |
| 5-10 |  |
| <5 |  |
| <5 | Less than 5 annually, but numbers vary |
| <5 | Less than 2 |
| <5 |  |
| >10 | Since 2018 majority of patients treated with contact brachytherapy |
| 5-10 | Dropped quite a bit since surgeons tend to do surgery and watchful waiting in N0 patients |
| >10 | 50 |
| >10 | 70 |
| <5 | At the moment we did not implement local rectal radiation treatment yet, however we are actively exploring both the brachytherapy and contact therapy |
| <5 | Around 5 |
| <5 | Previous employer, now wanting to start this indication |
| 5-10 | The number varies between 5-15. Since we have implemented endorectal high-dose-rate brachytherapy combined with EBRT for older/frail patients as an option for organ preservation, the number of patients has increased continuously. |
| >10 | CXB 40 patients annually-increasing to 80+ now HDR rectum 3-5 patients per annum |

3. At your institution brachytherapy is performed with the following intent

| **Answer** | **Comments** |
| --- | --- |
| Curative intent, palliative intent |  |
| Curative intent |  |
| Curative intent, palliative intent | radical treatment mainly |
| Curative intent |  |
| Palliative intent |  |
| Curative intent, palliative intent | Curative intent: dose escalation to achieve a complete response, palliative intent: unfit for surgery, symptomatic |
| Curative intent, palliative intent |  |
| Curative intent, palliative intent |  |
| Curative intent, palliative intent |  |
| Curative intent |  |
| Curative intent |  |
| Not applicable | Experience was gathered in both curative and palliative intent |
| Palliative intent | Mostly palliative; very occasionally curative |
| Curative intent |  |
| Curative intent, palliative intent | The majority of patients does receive treatment within the framework of an organ-preservation approach for non-metastatic disease in older/frail patients (non-surgical candidates). In addition, we offer treatment with palliative intent for multimodally pre-treated patients in the recurrence setting. |
| Curative intent, palliative intent | Generally CXB curative and HDR palliative-also goes on bulk |

4. Positive nodes are treated with brachytherapy

| **Answer** | **Comments** |
| --- | --- |
| Yes | In a palliative setting |
| No |  |
| No |  |
| No |  |
| No |  |
| No |  |
| No |  |
| No |  |
| No |  |
| Yes | Patients with positive nodes receive a boost dose as SIB during EBRT and if the primary tumor response is conducive, brachytherapy is given to achieve successful wait and watch. |
| No | No we treat with EBRT boost |
| No |  |
| No |  |
| Yes | With HDR BT, nearby nodes receive dose. However, it is not our intention to deliver a minimum dose to nodes with Brachy |
| No |  |
| No | Not specifically but if within 1cm of mucosa could be included |

5. Imaging modality at time of diagnosis

| **Answer** | **Comments** |
| --- | --- |
| Pelvic MRI, CT including pelvis, endorectal ultrasound | PET-CT is optional, in some patients we do it |
| Pelvic MRI, flexible endoscopy |  |
| Pelvic MRI, CT including pelvis, flexible endoscopy |  |
| Pelvic MRI, CT including pelvis, flexible endoscopy, PET-CT |  |
| Pelvic MRI, CT including pelvis, flexible endoscopy, PET-CT |  |
| Pelvic MRI, CT including pelvis, flexible endoscopy | PET CT is delivered in case of differential diagnosis between scar and local relapse in the pelvis, or for dubious nodules of at least 1 cm (i.e. lung, abdominal nodules). Abdomen MR in case of liver lesions |
| Pelvic MRI, CT including pelvis, flexible endoscopy |  |
| Pelvic MRI, CT including pelvis, flexible endoscopy |  |
| Pelvic MRI, CT including pelvis, flexible endoscopy, endorectal ultrasound, PET-CT |  |
| Pelvic MRI, flexible endoscopy, CECT chest and abdomen |  |
| Pelvic MRI | MRI pelvis and CECT thorax or abdomen |
| Pelvic MRI, CT including pelvis, flexible endoscopy | Patients with curative intent are usually with referred with diagnostic imaging performed at the referring institutions. We do take into account MRI and CT, however often do not possess many images of the endoscopy. However, if brachy and/or contact therapy will be implemented we would at least require more images of the endoscopy, and make use of rigid rectoscopy during treatment |
| Pelvic MRI, CT including pelvis, flexible endoscopy, rigid rectoscopy, PET-CT |  |
| Pelvic MRI, CT including pelvis, flexible endoscopy, endorectal ultrasound |  |
| Pelvic MRI, CT including pelvis, flexible endoscopy, rigid rectoscopy, endorectal ultrasound | Pelvic-MRI, endoscopy and ultrasound are mandatory at the time of diagnosis and prior to the first brachytherapy |
| Pelvic MRI, CT including pelvis, flexible endoscopy | EUS only for ?T1 patients to determine if brachy alone or TEMS |

6. In curative setting patients receive the following neoadjuvant therapy before brachytherapy

| **Answer** | **Comments** |
| --- | --- |
| Long-course radiotherapy (20 fractions or more), chemotherapy in combination EBRT |  |
| Medium-course EBRT | We use 13 x 3 Gy followed by 3 brachytherapy applications |
| Short-course EBRT (5 fractions or less), chemotherapy in combination EBRT | EBRT 5 x 5 Gy plus 3x FOLFOX every 2 weeks |
| Long-course radiotherapy (20 fractions or more), chemotherapy in combination EBRT |  |
| Not applicable |  |
| Short-course EBRT (5 fractions or less), chemotherapy in combination EBRT | 1. concomitant LC chemoradiation 2. TNT LC chemoradiation with consolidation chemotherapy 3. TNT SC RAPIDO like |
| Short-course EBRT (5 fractions or less), chemotherapy in combination EBRT |  |
| Short-course EBRT (5 fractions or less), Long-course radiotherapy (20 fractions or more) |  |
| Short-course EBRT (5 fractions or less), Long-course radiotherapy (20 fractions or more), chemotherapy in combination EBRT |  |
| Short-course EBRT (5 fractions or less), long-course radiotherapy (20 fractions or more), chemotherapy in combination EBRT | For LCRT: concurrent chemoradiation with capecitabine For SCRT: standard SCRT with or without SIB up to 30Gy/5# followed by 12-18 weeks of chemotherapy. Sometimes patients may receive some cycles of chemotherapy due to logistics in radiotherapy appointments. |
| Long-course radiotherapy (20 fractions or more) | Routinely 45-5-gy/25# + concurrent chemo SCRT + chemo in an ongoing study |
| Chemotherapy in combination EBRT, none (BT only) | Our idea would be 25x1.8-2 Gy followed by a 3-week break --> start of 3x10 Gy BT. In case of BT only 4x6.5 Gy in 4 consecutive days |
| Short-course EBRT (5 fractions or less), long-course radiotherapy (20 fractions or more), chemotherapy (no EBRT), chemotherapy in combination EBRT |  |
| Medium-course EBRT, chemotherapy in combination EBRT |  |
| Medium-course EBRT | Our concept is: 1) 13x3 Gy EBRT to the pelvis 2) 6 weeks after completion of EBRT re-staging with pelvic-MRI, endoscopy and ultrasound is being performed. During endoscopy the cranial, caudal and lateral borders of the remaining macroscopic tumor are marked with clips. 3) Brachytherapy starts the day after completion of re-stating with endoscopy being the last diagnostic procedure. HDR-afterloading is being performed once-weekly with overall 3 single-fractions procedures. |
| Short-course EBRT (5 fractions or less), chemotherapy in combination EBRT | SCRT if not fit for chemo. CRT long course if fit for chemo |

7. In palliative setting patients receive the following neoadjuvant therapy before brachytherapy

| **Answer** | **Comments** |
| --- | --- |
| Short-course EBRT (5 fractions or less), chemotherapy (no EBRT), none (BT only) |  |
| Not applicable |  |
| Short-course EBRT (5 fractions or less) | Rare cases |
| Not applicable |  |
| Short-course EBRT (5 fractions or less) | Some patients have SCRT, but some have brachy alone |
| Short-course EBRT (5 fractions or less) |  |
| Short-course EBRT (5 fractions or less) |  |
| Short-course EBRT (5 fractions or less) |  |
| Short-course EBRT (5 fractions or less), medium-course EBRT, long-course radiotherapy (20 fractions or more), chemotherapy (no EBRT), chemotherapy in combination with EBRT |  |
| Not applicable |  |
| Short-course EBRT (5 fractions or less) |  |
| Medium-course EBRT | Based on the HERBERT trial 13x3 followed by BT 6 weeks later |
| Short-course EBRT (5 fractions or less), long-course radiotherapy (20 fractions or more), chemotherapy (no EBRT), chemotherapy in combination EBRT |  |
| Not applicable |  |
| None (BT only) |  |
| Short-course EBRT (5 fractions or less), chemotherapy (no EBRT) |  |

8. In case of neoadjuvant therapy before BT, the following imaging modality is being used after neoadjuvant therapy

| **Answer** | **Comments** |
| --- | --- |
| Pelvic MRI, CT including pelvis, endorectal ultrasound |  |
| Pelvic MRI, flexible endoscopy |  |
| Flexible endoscopy |  |
| Pelvic MRI, flexible endoscopy |  |
| Pelvic MRI, CT including pelvis, flexible endoscopy |  |
| Pelvic MRI, CT including pelvis, rigid rectoscopy | MR is always delivered (if not contraindicated). Rigid rectoscopy is delivered in case of a major / complete clinical response. CT scan in case of MR cannot be delivered |
| Pelvic MRI, CT including pelvis, flexible endoscopy |  |
| Pelvic MRI, CT including pelvis, flexible endoscopy |  |
| Pelvic MRI, CT including pelvis, flexible endoscopy |  |
| Pelvic MRI | This is applicable for imaging at Brachytherapy. post brachy, assessment of response for Sx vs WnW, all 3 are done: MRI pelvis, Sigmoidoscopy, CECT chest and abdomen. |
| Pelvic MRI | MRI with applicator in situ |
| Pelvic MRI, flexible endoscopy, rigid rectoscopy |  |
| Pelvic MRI, CT including pelvis, flexible endoscopy, rigid rectoscopy, PET-CT |  |
| Pelvic MRI, flexible endoscopy, endorectal ultrasound |  |
| Pelvic MRI, CT including pelvis, flexible endoscopy, rigid rectoscopy, endorectal ultrasound, PET-CT |  |
| Pelvic MRI, CT including pelvis, flexible endoscopy |  |

9. Before brachytherapy, the position of (residual) tumor is marked

| **Answer** | **Comments** |
| --- | --- |
| Not marked |  |
| Using metal fiducial markers placed at flexible endoscopy |  |
| Using metal fiducial markers placed at flexible endoscopy, using metal fiducial markers placed at rigid rectoscopy |  |
| Using metal fiducial markers placed at flexible endoscopy |  |
| Fiducials placed during brachytherapy | Sometimes clips are placed during endoscopy before brachytherapy. If this does not happen we have placed fiducials to mark the tumour during the rigid sigmoidoscopy for brachytherapy |
| Using metal fiducial markers placed at flexible endoscopy | Four intramucosal MR-safe fiducials are placed in length and width directions |
| Using metal fiducial markers placed at flexible endoscopy |  |
| Using metal fiducial markers placed at flexible endoscopy |  |
| Using liquid fiducial markers placed at flexible endoscopy |  |
| Not marked | We use MRI for brachy planning |
| Not marked | We can see the residual tumor on MRI images |
| Using metal fiducial markers placed at flexible endoscopy |  |
| Using metal fiducial markers placed at rigid rectoscopy | we use a metallic seed inserted into the mucosa/sub mucosa |
| Using metal fiducial markers placed at flexible endoscopy |  |
| Using metal fiducial markers placed at flexible endoscopy |  |
| Using metal fiducial markers placed at flexible endoscopy | For HDR, CXB is direct visualisation |

10. Which imaging modality do you use for treatment planning of fraction 1 with the applicator in situ

| **Answer** | **Comments** |
| --- | --- |
| MRI, CT |  |
| CT |  |
| CT |  |
| MRI, CT | MRI and/or CT with markers + pre-BT MRI |
| CT |  |
| CT | Due to the markers placed, we use a CT scan |
| CT |  |
| CT |  |
| MRI, CT |  |
| MRI |  |
| MRI |  |
| CT | Currently in the institutions which we gained experience both used CT scan for this. However, ideally we would like an in-room CT/MRI for this |
| Plain film (X-ray) | We are developing a CT planning protocol |
| MRI, CT |  |
| CT | All 3 brachytherapy fractions are CT-based planned. We perform for every treatment fraction a CT-fusion with the previous planning-CT´s for reproducibility and plausibility control. |
| CT |  |

11. Do you plan and treat on the first fraction?

| **Answer** | **Comments** |
| --- | --- |
| Yes, every first fraction |  |
| Yes, every first fraction |  |
| Yes, every first fraction |  |
| Yes, every first fraction |  |
| No, we perform a pre-treatment planning scan | We have performed same-day treatments, but patients found the wait too long and uncomfortable so we no longer do this |
| Only for palliative cases |  |
| Only for palliative cases |  |
| Yes, every first fraction |  |
| Yes, every first fraction | Plan and treat at every fraction |
| Yes, every first fraction |  |
| Yes, every first fraction |  |
| Yes, every first fraction |  |
| Yes, every first fraction |  |
| Yes, every first fraction |  |
| Yes, every first fraction |  |
| Only for palliative cases | only for single fraction circumferential |

12. What imaging do you use for subsequent insertions?

| **Answer** | **Comments** |
| --- | --- |
| CT |  |
| CT |  |
| CT |  |
| MRI, CT | MRI or CT |
| CT |  |
| CT |  |
| CT |  |
| CT |  |
| MRI, CT |  |
| No imaging | same plan is delivered thrice |
| No imaging |  |
| CT | Currently in the institutions which we gained experience both used CT scan for this. However, ideally we would like an in-room CT/MRI for this |
| Plain film (X-ray) |  |
| CT |  |
| CT |  |
| CT |  |

13. Do you perform a new plan for every insertion?

| **Answer** | **Comments** |
| --- | --- |
| Yes |  |
| Yes |  |
| Yes |  |
| Only if significant change to GTV |  |
| Yes | The plan is generally kept the same but moved to correspond with the applicator position |
| Yes | In case of palliative cases and multiple subsequent fractions, the plan is re-performed in case of significant change of GTV |
| Yes |  |
| Yes |  |
| Yes |  |
| No |  |
| No |  |
| Yes |  |
| No |  |
| Yes |  |
| Yes |  |
| No |  |

14. The GTV is defined as:

| **Answer** | **Comments** |
| --- | --- |
| Tumor on CT at moment of brachytherapy |  |
| Tumor on MRI at moment of brachytherapy |  |
| Tumor on CT at moment of brachytherapy |  |
| Tumor on MRI at moment of brachytherapy |  |
| Tumor on CT at moment of brachytherapy |  |
| Tumor on MRI at moment of brachytherapy | And US-endoscopy |
| Tumor on MRI at moment of brachytherapy |  |
| Tumor on MRI at moment of brachytherapy |  |
| Tumor on CT at moment of brachytherapy |  |
| Tumor on MRI at moment of brachytherapy |  |
| Tumor on MRI at moment of brachytherapy | Residual tumor seen on MRI |
| Tumor on CT at moment of brachytherapy | which is adapted every fraction |
| Tumor on MRI at moment of brachytherapy |  |
| Tumor on MRI at moment of brachytherapy | Residual GTV: on MRI characteristics similar to GTV on MRI at diagnosis |
| Tumor on CT at moment of brachytherapy | Tumour at brachytherapy, i.e. volume within the fiducial area. MRI-pelvis prior to brachytherapy is being taken into consideration for anatomical orientation and tumor extension information. |
| Tumour on endoscopy using MRI and CT |  |

15. Regarding CTV definition as part of BT treatment preparation (*In case there are multiple CTV’s – for example CTV high risk and CTV low risk) please elaborate how each is defined in the next question*

| **Answer** | **Comments** |
| --- | --- |
| A fixed GTV-CTV margin is being used (please elaborate on margin below) | 5-10 mm GTV to CTV margin |
| CTV = GTV as defined in the last question |  |
| CTV = GTV as defined in the last question |  |
| CTV = GTV as defined in the last question |  |
| CTV = GTV as defined in the last question |  |
| A fixed GTV-CTV margin is being used, the CTV is the scarring after EBRT | CTV = (GTV + margin) + any visible scar  Margin: 1 cm in superior and lateral directions, 1 to 0 cm in inferior direction and 0 cm in the mesorectum |
| A fixed GTV-CTV margin is being used, the CTV is the scarring after EBRT |  |
| CTV = GTV as defined in the last question |  |
| The CTV is the scarring after EBRT |  |
| CTV = GTV as defined in the last question |  |
| CTV = GTV as defined in the last question | No CTV |
| CTV = GTV as defined in the last question, the CTV is the scarring after EBRT | In case of palliative treatment with pretreatment planning the CTV can be defined using scarring and macroscopic tumor on initial imaging |
| CTV = GTV as defined in the last question |  |
| CTV = GTV as defined in the last question, the CTV is the scarring after EBRT | CTV-HR is a composite contoured by the radiation oncologist consisting of residual GTV (MRI) and pathological tissue suspicious for harboring macroscopic disease (scarring?). this takes also findings on CT at BT including position of markers into account. CTV-IR takes initial tumor extension into account and has a minimal margin of 5mm from CTV-HR taking anatomical boundaries into account (based on risk of significant microscopic disease from macroscopic tumor on surgical specimens) CTV-LR low risk is EBRT volume |
| The CTV is the scarring after EBRT |  |
| CTV = GTV as defined in the last question |  |

16. PTV is delineated as part of BT treatment – *if applicable: Please elaborate on margins*

| **Answer** | **Comments** |
| --- | --- |
| No |  |
| No |  |
| No |  |
| No |  |
| Only if a single treatment plan is used for multiple fractions |  |
| No |  |
| No |  |
| Yes, routinely |  |
| No |  |
| No | Usually, when you cover GTV with 100% isodose, it has a 7-10 mm margin cut off on the rectal wall, with accounts for ITV-PTV concepts |
| Yes, routinely | 5mm |
| No |  |
| No |  |
| No |  |
| Yes, routinely | Our agreement with the department of endoscopy is the placement of fiducials at 8-10 mm beyond the macroscopic tumour borders. We do not delineate a PTV outside the area within the fiducial surface. However, tumor description is being performed in lithotomy position with precise information on lesion length and position (e.g. 3-6 o'clock at the left side). |
| Yes, routinely | 5mm sup inf, 2mm circumferentially because we use same plan |

17. Treatment intent (palliative v. curative) affects GTV/CTV/PTV delineation – *if yes, please elaborate on the impact and in what scenarios*

| **Answer** | **Comments** |
| --- | --- |
| Yes | In palliative setting it is usually GTV = CTV |
| Not applicable |  |
| No |  |
| Not applicable |  |
| No |  |
| Yes | CTV is not usually delineated in all palliative treatments. In case of tumor infiltrating other organs, and ERT is not deliverable, brachy can be delivered to a partial GTV |
| Yes | In palliative treatments CTV is not routinely contoured |
| No |  |
| No |  |
| Not applicable | we do not treat palliative BCT |
| No |  |
| Yes | In curative patients you would distinct especially between patients with planned organ preservation to make sure everything is covered. In palliative patients with no surgery you will delineate the CTV somewhat larger, however spare OARs more in advance |
| Not applicable |  |
| Not applicable |  |
| Not applicable |  |
| No |  |

18. The imaging modality used with applicator in situ affects GTV/CTV/PTV delineation – *if yes, please elaborate on the impact and in what scenarios*

| **Answer** | **Comments** |
| --- | --- |
| No |  |
| No |  |
| Not applicable |  |
| No |  |
| No |  |
| Yes | We delineate on CT scan, merged with diagnostic pelvic MR. If diagnostic pelvic MR is not available more generous GTV-CTV margins are applied |
| Yes | MRI fused to CT scan is used to delineate the contour |
| No |  |
| No |  |
| Yes | We use an applicator with double-balloon, which spreads the tumour dimensions wider and thinner |
| Yes | The GTV can be marked entirely |
| Yes | In case of an MRI a greater distinction between GTV and CTV would be possible |
| Yes | If CT or MRI is used, the GTV CTV PTV are likely to be different compared to plain x ray |
| Yes | If only CT at BT then no GTV delineation but directly CTV-HR |
| No |  |
| No |  |

19. Usage of markers affects GTV/CTV/PTV delineation – *if yes, please elaborate on the impact and in what scenarios*

| **Answer** | **Comments** |
| --- | --- |
| Not applicable |  |
| Yes | Sometimes marker position seems a bit different from what is expected from notes at placement. In that case a consensus on delineation is reached with a colleague |
| Not applicable |  |
| Yes | Depending on endoscopic position of markers. Usually markers = border of GTV |
| No |  |
| Yes | Metal markers generate artefacts slightly affecting the visibility of the target on CT scan, although we always delineated on merged MR CT images |
| Yes | Metal markers typically bring about some minor artefacts |
| No |  |
| Yes | Guide us to correlate to GTV seen with rectoscope |
| Not applicable | We do not use marker |
| Not applicable |  |
| No |  |
| Yes | It is likely to be much more |
| Yes | This depends on location of clips as per placement endoscopically and imaging. But position of clips is taken into account when delineating CTV |
| No |  |
| No |  |

20. Relationship to (specific) OARS affects GTV/CTV/PTV delineation – *if yes, please elaborate on the impact and in what scenarios*

| **Answer** | **Comments** |
| --- | --- |
| No |  |
| No |  |
| No |  |
| No |  |
| No |  |
| Yes | A catheter is positioned to have bladder filling under control. Anterior cancer in the lower pelvis may impact vagina doses, in this case, to avoid fistula, dose distribution must be carefully directed |
| Yes | We pay the highest degree of attention in order to keep the dose to OARs as low as possible |
| No |  |
| No |  |
| Yes | On MRI, delineation near the sphincter complex gets tricky |
| Not applicable |  |
| No | Not so much the delineation, maybe more the planning |
| Not applicable |  |
| No |  |
| No |  |
| No |  |

21. Post-brachytherapy treatment options (e.g. potential watch & wait vs planned TME surgery) affect GTV/CTV/PTV delineation – *if yes, please elaborate on the impact and in what scenarios*

| **Answer** | **Comments** |
| --- | --- |
| No |  |
| Not applicable |  |
| No |  |
| No |  |
| No |  |
| No | Usually, GTV/CTV delineation is not affected by post-BT treatment options, even though in case of WW, volumes should be carefully reviewed to avoid unwanted missing. |
| No |  |
| No |  |
| Yes | MRI 4 weeks later |
| No |  |
| No |  |
| Yes | In two ways, one you want to make sure you entirely cover any suspicious tissue in order to planned W&W. In case no surgery follows you also want to spare the contralateral wall even more |
| Not applicable |  |
| No |  |
| No |  |
| No |  |

22. Other factors (not mentioned yet) affect GTV/CTV delineation – *if yes, please elaborate on the impact and in what scenarios*

| **Answer** | **Comments** |
| --- | --- |
| Yes | If it is a reirradiation because of the recurrence usually GTV= CTV |
| No |  |
| No |  |
| No |  |
| No |  |
| No |  |
| No |  |
| No |  |
| No |  |
| Yes | Trapped gas around applicator, enema fluid, tumour/rectal wall oedema, dense post treatment fibrosis |
| Yes | Rectal filling |
| No |  |
| Not applicable |  |
| No |  |
| No |  |
| No |  |

23. Institutional guidelines/protocols regarding target volume delineation are available at my institution

| **Answer** | **Comments** |
| --- | --- |
| No |  |
| Yes | Those are the protocols for the HERBERT 1 and 2 study |
| Yes |  |
| No |  |
| Yes | 7.1 The CTV is contoured 7.2 The ‘grip end’ clip position is defined as where the clip attaches to the bowel wall. 7.3 Consider contouring small bowel as OAR for high tumours 7.4 The first dwell position (FDP) is defined by physics, this will be 0.5-1 cm above the CTV. 7.5 Regions of interest • Clip 1 grip end • Clip 1 • Clip 2 grip end • Clip 2 • Clip 3 grip end • Clip 3 • FDP • PTV |
| No |  |
| No |  |
| No |  |
| No |  |
| Yes | MRI t2 image residual tumour (hyperintense) with dark fibrotic change in concurrence with clinical findings at Brachytherapy |
| No |  |
| No |  |
| Yes | Local guidelines available |
| No |  |
| Yes |  |
| Yes | not much on target volume, more about who/what to treat |

24. OARs should be contoured in my institution

| **Answer** | **Comments** |
| --- | --- |
| Yes | **Routinely**: bladder, urethra, sigmoid, non-rectal bowel, anal canal |
| Yes | **Routinely**: contralateral bowel mucosa, bladder, anal canal  **Only as part of a research protocol**: urethra, vagina, non-rectal bowel |
| Yes | **Routinely**: contralateral bowel mucosa, anal canal |
| Yes | **Routinely**: contralateral bowel mucosa, bladder, urethra, vagina, non-rectal bowel, anal canal  **Only as part of a research protocol**: penile bulb, vulva, sacrum, sigmoid, perianal skin |
| Yes | **Routinely**: bladder (if it is within 2cm of the CTV) |
| No |  |
| No |  |
| No |  |
| Yes | **Only as part of a research protocol**: contralateral bowel mucosa |
| Yes | **Only as part of a research protocol**: contralateral bowel mucosa (we started recently), bladder (posterior wall started recently), urethra (may not be necessary), prostate (may not be required), penile bulb (started recently for tumour in that zone in males), vagina (started contouring recently in the region of tumor), anal canal (maybe only if in more than 50% isodose?) |
| Yes | **Routinely**: urethra  **Only as part of a research protocol**: contralateral bowel mucosa, bladder, prostate, penile bulb, vagina, vulva, sacrum, sigmoid, non-rectal bowel, anal canal, perianal skin |
| No |  |
| No |  |
| Yes | **Routinely**: contralateral bowel mucosa, bladder, anal canal  **Only as part of a research protocol**: urethra, prostate, penile bulb, vagina, vulva, sacrum, sigmoid, non-rectal bowel, perianal skin (all depending on tumor location) |
| Yes | **Routinely**: contralateral bowel mucosa (We delineate healthy mucosa at least 30 mm cranial and caudal of the PTV as well as contralateral mucosa), bladder (We delineate the bladder for treatment planning. All patients receive a Foley catheter with 50 ml contrast dye solution prior to the treatment planning CT. Consecutively the bladder is emptied and 50 ml NaCl injected prior to HDR irradiation.), urethra (We delineate the urethra for treatment planning. All patients receive a Foley catheter and the urethra can be identified.), prostate (We delineate the prostate for treatment planning), anal canal  **Only as part of a research protocol**: penile bulb, vagina, vulva, sacrum, sigmoid (Sigmoid delineation results in relation to the primary tumor location. However, I have not treated many tumors of the upper rectum with brachytherapy so far.), non-rectal bowel, perianal skin |
| Yes | **Routinely**: bladder, urethra, sacrum, non-rectal bowel, anal canal (all if within 2cm of GTV)  **Only as part of a research protocol**: contralateral bowel mucosa (Try to but no good consensus what it is), |

25. How do you determine which OAR to contour?

| **Answer** | **Comments** |
| --- | --- |
| Contour all within a certain distance superior and inferior to GTV/PTV | In diameter at least 3 cm around CTV |
| Contour all within a certain distance superior and inferior to GTV/PTV |  |
| Expand GTV by a certain distance and contour within that | 2 cm distal and proximal |
| Contour all within a certain distance superior and inferior to GTV/PTV |  |
| Contour all within a certain distance superior and inferior to GTV/PTV | 2cm |
| Not applicable |  |
| Not applicable |  |
| Not applicable |  |
| Contour all within a certain distance superior and inferior to GTV/PTV |  |
| Contour all within a certain distance superior and inferior to GTV/PTV | 2 cm zone/50% isodose region, which ever is more |
| Contour all within a certain distance superior and inferior to GTV/PTV |  |
| Not applicable |  |
| Not applicable |  |
| Expand GTV by a certain distance and contour within that | at least all OAR within 2cm of CTV |
| Everything seen on the planning CT | We delineate healthy mucosa at least 30 mm cranial and caudal of the PTV as well as contralateral mucosa. In addition we delineate prostate, urethra, anal canal (i.e. sphincter) as volumes within the planning-CT. |
| Expand GTV by a certain distance and contour within that | 2cm expansion |

26. Institutional guidelines/protocols regarding OAR delineation are available at my institution

| **Answer** | **Comments** |
| --- | --- |
| No |  |
| Yes | From HERBERT 1 and 2 study |
| No |  |
| No |  |
| No |  |
| Not applicable |  |
| Not applicable |  |
| Not applicable |  |
| No |  |
| Yes | Recently started as a study |
| No |  |
| Not applicable |  |
| Not applicable |  |
| No |  |
| No |  |
| No |  |
